# Geographical discrimination of palm oils (*Elaeis guineensis*) using quality characteristics and UV‐visible spectroscopy

**DOI:** 10.1002/fsn3.614

**Published:** 2018-03-09

**Authors:** Olusola S. Jolayemi, Mary A. Ajatta, Abimbola A. Adegeye

**Affiliations:** ^1^ Department of Food Science and Technology The Federal University of Technology Akure Nigeria

**Keywords:** OPLS‐DA, palm oils, PCA, quality parameters, UV‐visible spectroscopy

## Abstract

This preliminary study demonstrated the possibility of discriminating geographical origin of palm oils using conventional quality characteristics and UV‐visible spectroscopy. A total of 60 samples, 20 from each region (North (N), South (S), and Central (C)) of Ondo State Nigeria, were analyzed for their quality characteristics and UV‐visible spectra. Principal component analysis (PCA) and orthogonal projection to latent structure discriminant analysis (OPLS‐DA) were applied to elaborate the data. Models were built on the most informative portion of the spectra (250–550 nm) as: untreated (without pretreatment) and standard normal variate—second‐derivative‐treated (SNV+2der) data matrices. OPLS‐DA classification models were validated by independent prediction sets and cross‐validation. PCA score plots of both chemical and spectral data matrices revealed geographical distinction between the palm oil samples. Significantly high carotene content, free fatty acids, acid value, and peroxide value distinguished Central palm oils. K extinction values, color density, and chlorophyll content were the most important quality parameters separating North oil samples. In the discriminant models, over 95% and 85% percent correct classification were recorded for spectral and chemical data, respectively. These results cannot be considered exhaustive because of the limited sample size used. However, the study suggested a potential analytical technique suitable for geographical origin authentication of palm oils with additional advantages that include the following: speed, low cost, and minimal waste.

## 1 INTRODUCTION

The economic and nutritional contributions of palm oil to the World's oils and fats industries are highly significant; exceeding soya oil by a wide margin (Mancini et al., 2015). Its global popularity is as a result of a number of competitive advantages over other vegetable oils. These include low cost of production, modifiable chemical composition, and suitability in various food applications (Mba, Dumont, & Ngadi, 2015). Nutritionally, palm oil is relatively high in saturated fatty acid counterbalanced with monounsaturated and polyunsaturated in addition to other important minor bioactive phytonutrients (Odia, Ofori, & Maduka, 2015). These minor compounds include the following: carotenoids, tocopherols, chlorophyll, sterols, squalene, phospholipids, and about 1% polyphenols (Mba, Adewale, Dumont, & Ngadi, 2014). These chemical parameters vary with geographical origins of palm fruits from which the oil is obtained. Free fatty acids (FFAs), oil content, and maturation index of palm fruits are the three major parameters commonly used to predict palm oil quality (Makky, 2016). These parameters are not comprehensive enough to sufficiently describe all the intrinsic differences between palm oils produced from different regions. It is a well‐known fact that combination of analytical fingerprinting and multivariate data evaluation could facilitate nontargeted class differentiation between food products (Alewijn, van der Voet, & van Ruth, 2016). These methods create specific patterns that might be based on chemical compositions, geographical origin, and other distinctive variables peculiar to the product (Tres & Van Ruth, 2011). Spectral or chromatograms obtained as response of certain analytical equipment can provide useful information about food product, that would be unnoticed by the use of conventional approaches (Bosque‐Sendra, Cuadros‐Rodríguez, Ruiz‐Samblás, & de la Mata, 2012).

Like every other agricultural produce, variables influencing the chemical characteristics of palm oils include the following: geographical location, cultivar, agronomic practice, and production method. The meaning of origin is not only limited to provenance, it involves prevailing natural factors, cultural practices, and other historical attributes contributing to the relationship between food and place (William & Jen, 2017). This relationship links the quality attributes of the product with its geographical location. For example, the quality characteristics of some vegetable oils have been vastly linked with their regions of production (Karabagias et al., 2013; Tres, Ruiz‐samblas, van der Veer, & van Ruth, 2013; Uncu & Ozen, 2016). Generally, products obtained from regions known for higher desirable quality attributes have better market reputation. Information on these specific attributes peculiar to palm oils obtained from such regions could influence consumers' preference and perception. Consumers are gradually becoming keen in their willingness to pay slightly higher price for better quality when properly informed (García‐González & Aparicio, 2010). Therefore, regions reputed for better quality palm oils are likely to attract higher market share both locally and abroad. In addition, the subject of “sustainable palm oil production” is becoming a global issue. The initiative is targeted toward protecting palm oil‐producing areas from negative environmental impacts. Products from geographical areas certified for sustainable palm oil production automatically enjoy premium market (Fitzherbert et al., 2008). Presently, only inspection and administrative controls are used to identify sustainable palm oils (Tres et al., 2013). In order to be more objective in identifying sustainable palm oil, a more rapid analytical method would be an added advantage.

Spectroscopy is one of the most popular and highly adaptable techniques in conventional food analysis. UV‐visible region of electromagnetic radiation offers important advantages such as: direct measurement with little or no prior sample preparation, low cost of equipment, time‐saving, low manual intervention, and small sample required. So far, information on geographical origin authentication of palm oils is relatively low (Osorio, Haughey, Elliott, & Koidis, 2014). There are a few studies focusing on the application of one or more regions of electromagnetic spectrum in palm oil characterization. Mba et al. (2014) characterized the binary blends of palm and canola oils using NIR. Similarly, the potentials of near‐infrared spectroscopy in adulterants detection and quality authentication of palm oils were evaluated with satisfactory results (Basri et al., 2017; Mba et al., 2014). Moreover, there are a few other studies on chromatographic and spectroscopic determination of either minor or major components of palm oil in the literature with remarkable results (Azeman et al., 2015; Che Man, Aye, Tan, & Abdukarim, 2009; Moh et al., 1999). Apart from a recent study that predicted geographical origin of palm oil using HPLC (Obisesan, Jiménez‐Carvelo, Cuadros‐Rodriguez, Ruisánchez, & Callao, 2017), there is no study in the literature where geographical origin of palm oil is discriminated using UV‐visible spectroscopy. Therefore, the objective of this study was to show the possibility of differentiating palm oils produced within the same state into regions using their quality characteristics, UV‐visible spectral in conjunction with classical multivariate data elaboration.

## 2 MATERIALS AND METHODS

### 2.1 Palm oil samples

Sixty crude palm oil samples obtained from three regions of Ondo State Nigeria (North, Central, and South) were evaluated. Twenty samples from each region and the samples were collected from four different production mills under semimechanized production processes. Samples obtained from Ile‐Oluji/Okeigbo area constitute the South (S), and those from Akungba as North (N), and that of Akure as Central (C). These geographical locations are comparatively small compared to the entire production regions in the country, but constitute the main production sites for the entire south‐western part of the country. The samples were collected immediately after production, kept in dark glass bottles, and stored in a cool dry place prior to analysis.

### 2.2 Chemical analysis

#### 2.2.1 Free fatty acids, acid value, peroxide value, and K‐specific extinction coefficients determinations

According to official methods of American Oil Chemist' Society (AOCS, 1990), FFA, acid value (AV), peroxide value (PV), and K values were measured as average of three analyses per sample. Free acidity (0.503AV) indicative of the fatty acid content, expressed as oleic acid (%), was determined by titrating the oil solution (ethanol:ethyl ether, 1:1) with 0.1 N KOH, and phenolphthalein was used as indicator. PV expressed as equivalents of active oxygen per kg of oil (meqO_2_/kg) was determined by the reaction of oil mixture (chloroform, acetic acid, and palm oil) with potassium iodide in the absence of light. The iodine liberated was titrated with 0.1 N sodium thiosulfate solution using 1% starch solution as an indicator. Spectrophotometric indices, also known as specific extinction coefficients, *K*_270_ and *K*_232_ and ΔK were measured as the absorption values of oil solution (cyclohexane and palm oil) at 232 and 270 nm wavelengths, respectively, using UV‐vis spectrophotometer (Shimadzu UV‐1800 Kyoto, Japan) with 1‐cm path length.

#### 2.2.2 Chlorophyll and carotenoid determination

Modified method of Harborne (1980) was used for the determination of chlorophyll and carotenoid contents of the samples. Palm oil sample (100 mg) was mixed with 10 ml of 80% acetone, and the mixture was centrifuged at 1107 × g for 10 min. The supernatant was made up to 10 ml using 80% ethanol. The optical intensity (absorbance) was taken at 480 nm for carotenoids, at 645 nm, and 652 nm for chlorophyll in UV‐vis spectrophotometer (Shimadzu UV‐1800, Kyoto, Japan). Total chlorophyll and carotenoid contents were estimated using the equations below:

$$\text{Total carotenoid content (mg/kg)}=\frac{\left[4*A_{480\;\mathrm{nm}}*V*1,000\right]}{\text{Sample weight}}$$

$$\begin{array}{c} \text{Total chlorophyll content (mg/kg)}=\frac{\left[20.2*\left(A_{645\;\mathrm{nm}}\right)+8.02*\left(A_{663\;\mathrm{nm}}\right)*V\right]}{1,000*W} \end{array}$$

where *A*: absorbance of specific wavelength, *V*: final volume of chlorophyll extract in 80% acetone, *W*: weight of the oil sample.

#### 2.2.3 Color density

Spectroscopic method described by Wroistad (1993) was used in the color determination. Palm oil sample (1 ml) was diluted with 25 ml methanol in a beaker and stirred for 30 min using magnetic stirrer to enable proper color extraction. The mixture was allowed to stand for 10 min and centrifuged. Optical density or absorbance of the supernatant was taken at 420 and 520 nm wavelength using UV‐vis spectrophotometer (Shimadzu UV‐1800, Kyoto, Japan). The analyses were performed in triplicate. Color density was recorded as the sum of the absorbances of the two wavelengths thus:

$$\text{Color density}=A_{420\;\mathrm{nm}}+A_{520\;\mathrm{nm}}.$$

### 2.3 UV‐visible spectra acquisition

UV‐visible spectrophotometer (Shimadzu UV‐1800, Kyoto, Japan) equipped with deuterium‐discharge lamp as ultraviolet range source and a tungsten lamp for the visible with 2.0 nm resolution was used for the UV‐vis spectrum of the oil samples. There were two rectangular cells, one for sample (1 ml palm oil dissolved in 3 ml hexane) and the other for blank (pure n‐hexane). Quartz cuvette of 10‐mm path length was used for sample and blank holder as both soda and pyrex glass absorbed below 365 and 320 nm, respectively. The UV‐vis spectra of the samples taken between 200 and 800 nm with 2.0 nm equally spaced wavelength constitute the spectral data matrix.

### 2.4 Data processing and analysis

The significance of geographical differences between the oil samples with respect to their chemical parameters was determined by one‐way analysis of variance (ANOVA) at 95% confidence level (Minitab 16.0, Minitab Inc., State College, USA). In the multivariate analysis, calibration and validation models were prepared in two categories:

1. Chemical data matrix (60 × 11) consisted of 60 palm oil samples (*n* observations) and 11 measured variables (*K* variables). The variables involved are as follows: FFAs, chlorophyll and carotene, peroxide and acid values, *K* extinction (*K*_232_ and *K*_270_), and *R*‐value (*K*_232_/*K*_270_)
2. Spectral data matrix composed of reduced spectral range (250–550 nm) measured with two wavelength interval. The spectra segments with low signal‐to‐noise ratio (200–250 and 550–800 nm) were excluded from the matrix.

Noise and large variabilities usually common to spectroscopic data were removed by preliminary filtering techniques. Combination of standard normal variate (SNV) and second‐order derivatives (2der) was applied on the averaged spectra before calibration and validation models were developed. Ability of these pretreatments to separate scattered light from absorbed light has been previously verified (Jolayemi, Tokatli, Buratti, & Alamprese, 2017). SNV algorithm is a row‐oriented spectra pretreatment method that corrects baseline and removes noise using mean centering (Zeng, Huang, Xu, Ma, & Wu, 2016). First‐ and second‐derivatives with 15 points smoothing gap (Savitzky‐Golay polynomial) distance correct spectral perturbation, noise, and increase signal‐to‐noise ratio (Xu et al., 2008). The most widely applied linear chemometric techniques are the unsupervised principal component analysis (PCA). It is a trend, pattern, and outlier recognition method that linearly transform data matrix. The transformation leads to the maximum preservation of as many variance in the original data as possible in lower dimensionality space called principal components (PC) (Worley & Powers, 2013). This linear data decomposition facilitates simpler and unbiased interpretation of the datasets.

Calibration and validation models were built using OPLS‐DA (orthogonal projection to latent structure discriminant analysis). The technique depends on previously defined membership class information (**Y**) of each observation (palm oil) relative to the chemical and spectral data **X** matrices. The class memberships were coded in the matrix form of **Y** as thus: class 1 (Central), class 2 (North), and class 3 (South) based on the oil regional differences. It is worthy of note to state that the same class specification was used for both spectral and chemical data matrices prior to class prediction. However, OPLS‐DA modifies the classical PLS‐DA with the incorporation of an inbuilt orthogonal signal correction filter that enables effective separation of **X** variations into **Y**‐predictive (related to class information) and **Y**‐uncorrelated (orthogonal or unrelated to class information) (Worley & Powers, 2013).

The same randomly selected external validation sets consisting of 15 oil samples (5 C, 5 N, and 5 S) were used to verify the model's predictiveness in both chemical and spectral data. In addition to this, inbuilt cross‐validation method with seven cancellation groups (7 CV) was performed to further verify the robustness of the models. SIMCA software (v. 13 Umerics, Umea, Sweden) was used for all the multivariate statistical analyses, and the output parameters were recorded. These parameters include the number of significant PCs used (PC_p + PC_o where p and o represent predictive and orthogonal components, respectively) in the case of OPLS‐DA, determination coefficient for calibration ($R_{\mathrm{cal}}^{2}$), cross‐validation ($R_{\mathrm{cv}}^{2}$), and confusion matrices (percentage correctly classified sample) for calibration and validation in OPLS regional discrimination of the oil samples.

## 3 RESULTS AND DISCUSSION

### 3.1 Chemical parameters

Table 1 shows the results of the chemical and quality characteristics of the oil samples. The acidity of the oils from the three regions considered varied between 6.71% and 9.52%, which is slightly outside the expected value (≤5.00%) for crude palm oil according to PORAM (2013) and CODEX 210 (2011) quality assessment criteria. Oxidation of unsaturated fatty acids is the main reaction responsible for the degradation of lipids and this forms the basis for the analytical quality assessment of palm oils. PV measures the extent of accumulation of primary oxidative product called “hydroperoxides” which has not actually been converted to secondary products responsible for actual deterioration of the oils and fats. Therefore, palm oils samples of higher PV (>4.60 meqO_2_/kg) may not necessary be of low quality, but suggest low oxidative stability of the C palm oils. High PV of C oils may be an evidence of prolonged time lag between harvesting and processing of palm fruits. However, all the palm oil samples were within acceptable minimum level (15 meq O_2_/kg) by CODEX 210 (2011). Oils are mixture of triacylglycerols that can be hydrolyzed enzymatically or chemically to generate a mixture of FFA, glycerol, mono, and diacylglycerols. The factors that mostly influence the rate of these reactions are related to environmental and processing conditions such as high temperature, moisture and oxygen availability, and exposed surface area (Choe & Min, 2007). These rate‐determining factors cannot be completely controlled in semimechanized palm oil extraction process. Therefore, the comparatively high FFA values and AV of the samples may be a reflection of the difference between industrial and semi‐intensive processing environments as earlier observed by De Almeida et al. (2013).

**Table 1:** Quality variation in palm oils based on regional differences

Quality parameters	Regions		
	Central	North	South
Free fatty acid (%)	8.98 ± 0.54^a^	8.17 ± 0.20^b^	7.12 ± 0.41^c^
Acid value (%)	17.96 ± 1.08^a^	16.34 ± 0.40^b^	14.24 ± 0.82^c^
Peroxide value (meqO_2_/kg)	3.97 ± 0.71^a^	2.23 ± 0.38^b^	0.99 ± 0.15^c^
*K*_232 nm_	0.27 ± 0.04^a^	0.27 ± 0.05^a^	0.25 ± 0.04^a^
*K*_270 nm_	0.16 ± 0.07^a^	0.18 ± 0.03^a^	0.09 ± 0.02^b^
Δ*K*	0.12 ± 0.06^a^	0.14 ± 0.03^a^	0.05 ± 0.02^b^
*R*‐value	1.95 ± 0.75^b^	1.53 ± 0.22^b^	2.85 ± 0.80^a^
Carotene (mg/kg)	737.83 ± 53.49^a^	608.80 ± 42.42^b^	501.70 ± 17.56^c^
Chlorophyll (mg/kg)	0.08 ± 0.02^b^	0.20 ± 0.07^a^	0.03 ± 0.01^c^
Color density	2.04 ± 0.58^b^	2.44 ± 0.54^a^	2.03 ± 0.39^b^

Means that do not share a letter (superscript) are significantly different at *p* ≤ .05.

The specific absorptions at 232 nm (*K*_232_) and 270 nm (*K*_270_) are related to the content of conjugated dienes and trienes compounds present in oils, respectively. *K* values are useful tool in providing a quick readout for oils quality comparison, but it does not provide information on the actual polyunsaturated fatty acids responsible for the diene and triene compounds. Therefore, apart from FFA and AV, there was no significant correlation between any pair of PV, FFA, and K values in establishing for facts, the impact of regional differences on the quality characteristics of the palm oil samples. However, free acidity values (FFA and AV) and PV of Central oil samples were significantly higher compared to other regions (Table 1). On the contrary, there was no significant regional influence on *K*_232_ while, *K*_270_, ΔK, and *R*‐value were the same for North and Central oil samples. Low *R*‐value and high *K*_270_ indicate the presence of more secondary oxidation products in the oils than primary (Multon, 1997). Therefore, oils from the North and Central regions are more susceptible to oxidative rancidity. The most widely distributed pigments present in palm oil are carotene with over 60% of it being beta‐carotene with potential vitamin A precursor and high radical scavenging capacity (Rufino et al., 2010). Apart from the nutritional importance of this pigment, it contributes to the visual appeal of palm oil and may influence the degree of consumer acceptability (Moyano, Heredia, & Melendez‐Martinez, 2010). Significantly, higher carotene content was obtained for palm oils from the Central region and followed by North. However, all the samples were within the minimum amount of carotene required for high‐quality unbleached palm oils (500–2,000 mg/kg) (CODEX 210, 2011). The variation in these values may be due to agronomical factors such as fruit cultivars, climatic conditions, and extraction procedures. Chlorophyll contents of the oil samples were relatively low with North samples having the highest chlorophyll content (0.20–0.34 mg/kg). The same trend was observed for color density as well.

### 3.2 UV‐visible spectra interpretation

**Figure 1:**
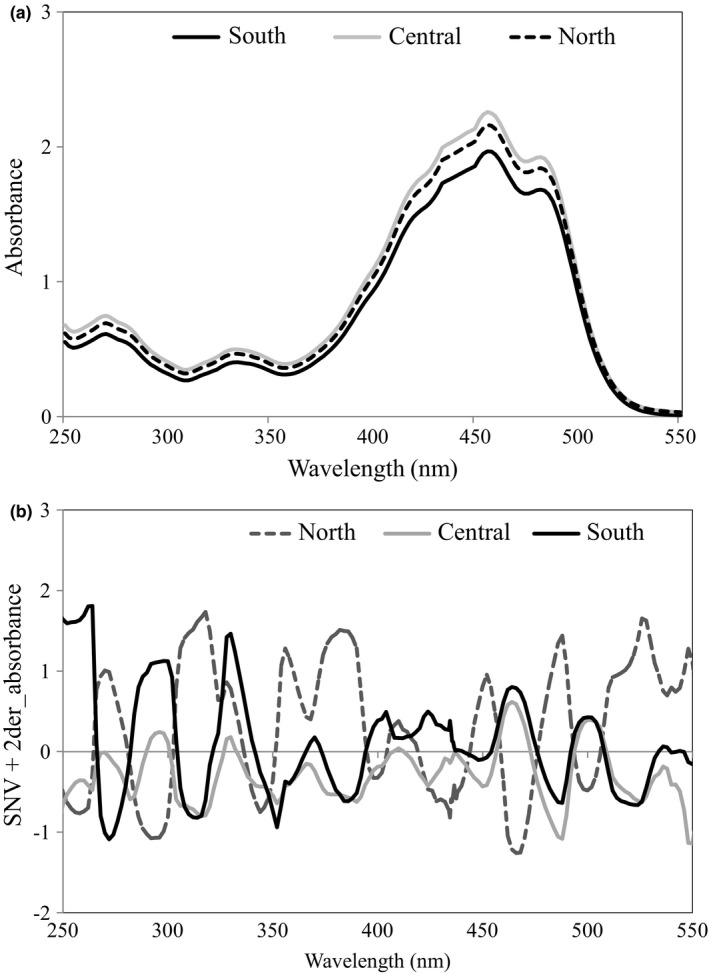
Illustration of UV‐vis spectra of palm oils: (a) Untreated and (b) SNV+2der‐treated spectra of palm oils of different regions (Central, North, and South)

The most significantly strong absorbance as shown in the raw spectra of the oil samples (Figure 1a) is between 260–320, 320–380, and 400–500 nm. These are all due to π electronic transitions that commonly provide information on the presence of conjugated unsaturations, conjugated nonbonding electron system, and aromatic compounds (Spatari, De Luca, Ioele, & Ragno, 2017). A single absorption band represented between 230 and 260 nm could be an indication of the presence of a number of compounds such as: cholesterol and some acyclic dienes, methylene‐interrupted and‐conjugate dienes and trienes, simple phenols, an aromatic amino acid and the likes (Pomeranz & Meloan, 1994) owing to the lipid nature of the samples. However, the little absorptions at 260–320 and 320–380 nm preceding the broadband of 400–500 nm showed unequivocally that the broadband is a member of carotenoid; the most chromogenic pigments present in palm oil (Ngomo, Mbah, Kamga, & Dinica, 2016). Beta‐carotene is the predominant form of the pigment in palm oil with major influence on its regional differences as shown in the chemical data. Furthermore, other probable compounds with certain degree of absorption properties include unsaturated compounds (particularly the polyacetylenes, those of aromatic origin, and ketones. Although there are no significant absorption bands between 500 and 800 nm (not shown), this region may provide some useful information on possible chemical reactions or changes in the palm oil. For instance, a meaningful absorption at this region has been shown to correspond to an equivalent decrease in carotene band, especially when acidity of the matrix is high (Boon, McClements, Weiss, & Decker, 2010). Dissolution of palm oil in organic solvent before taking the spectra may have deprived the spectra from revealing some saturated lipid fractions and organic acids especially between 500 and 800 nm. The plots of filtered spectra showed some variations along regions of informative bands (Figure 1b). The removal of noise and baseline tilting using SNV and 2der improved the spectra differential quality.

### 3.3 Multivariate data evaluation

#### 3.3.1 Principal component analysis (PCA)

**Figure 2:**
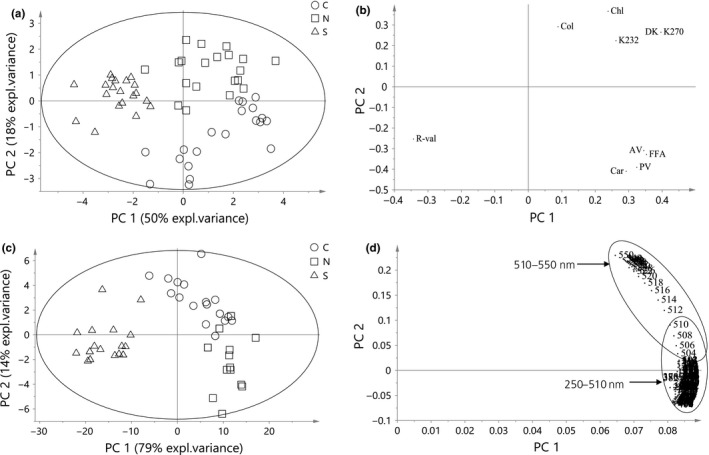
PCA model results: (a) score plot of quality parameters (b) loading plot of quality parameters (c) score plot of untreated spectral data, and (d) Loading plot of untreated spectral data of palm oils of different regions (Central C, North N, and South S)

Separate PCA models were built for quality parameters, untreated, and SNV+2der‐treated spectral data matrices in an attempt to determine possible trend among the oil samples (Figure 2). PCA model of quality parameters data with 3 PC and 79% total explained variance produced three clusters based on regional differences between the palm oil samples with S oils completely distinguished from others, forming a distinct cluster on the left side of control eclipse (Figure 2a). The variable most responsible for the separations of S oils as revealed in the loading plot is R‐value indicative of how distinctive the region is compared to N and C (Figure 2b). Even though maximum class separation is not the explicit objective of PCA, a close to perfect class separation was obtained from the score plots of both chemical and spectral data matrices. A slight overlap was observed between N and C at the positive axis of the PC 2 in both chemical and spectral data probably indicating some chemical similarities. High values of K values, chlorophyll, and color density helped to describe the projections of N palm oils while; carotene, acidity, and PVs were responsible for the clustering of C oils. However, untreated spectral data of the oil samples generated a PCA model with more descriptive explained variance of 99% with 6 PC. The first two PCs explained 93% total variance significantly higher than that of chemical data. The score plots output of the two data matrices looked visually similar (Figure 2a,c) with S palm oils clearly separated. The information embedded in the spectra regions influencing separate clusters cannot be strongly ascertained, but indicate nonspecific regional variations among the oil samples. As regards the spectral data, the most significant wavelengths responsible for the projection of observations on the score plane were shown (Figure 2d). These wavelengths are located on the positive axis of the first PC. High absorbance values of 250–510 nm range were responsible for the separation of N palm oils. Similarly, 510–550 nm spectra range was the most defining band separating C palm oils. However, S palm oils have comparatively lower absorbances in both spectral ranges. The slight similarity between some Central and North palm oil samples was evident within 500–510 nm spectral range. Similar patterns with slight difference were obtained for the PCA score plots of SNV+2der‐treated data (not shown). The information conveyed by PCA creates the basis by which the secondary discriminant analysis OPLS is validated as will be shown later.

#### 3.3.2 OPLS discriminant analysis

**Figure 3:**
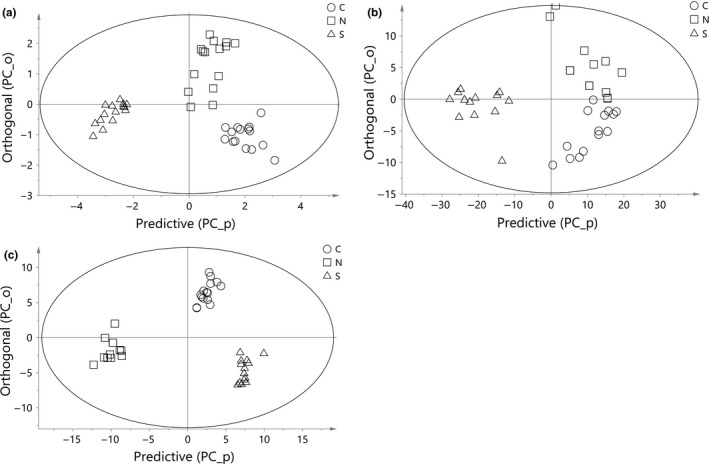
OPLS‐DA calibration model score plots: (a) quality parameters (b) untreated spectra, and (c) SNV+2der‐treated spectra of palm oils at different regions (Central C, North N, and South S)

Being a natural exploratory analysis, PCA shows a good distinction, but cannot be used for classification of samples into their geographical regions. Thus, class‐modeling technique that allows initial allocation of samples into classes prior to modeling is required. Therefore, in order to predict correct regional classes of palm oils using chemical, untreated spectra and SNV+2der‐treated spectral data matrices, OPLS discriminant method was adopted. The performance of the method in the data matrices was compared in terms of percentage of correct classification (Table 2) and coefficients of determination (Table 3). Both chemical and spectra models were fitted for training (calibration) and prediction (validation), and their calibrations score plots were presented in Figure 3. The differences in OPLS‐DA performances of the chemical and spectral data were significantly apparent in both the score plots and confusion tables especially when chemical data were compared to treated spectra (Figure 3b,c). There was a complete resolution of palm oils regional class overlapping especially in the predictive direction, as a result of separation of orthogonal variation to improve the discriminatory capacity of OPLS‐DA. This inbuilt error‐filtering advantage improves class‐modeling ability of OPLS‐DA (Bylesjo et al., 2006). The calibration and validation models of each class of palm oils indicated an average of over 90% correct regional prediction in spectral data which is slightly higher than that of chemical parameters. As observed in all of the data matrices, there was no misclassified S oil sample in both calibration and validation models of the spectral data. Conversely, in the chemical data, three N samples were misclassified into C class; two in calibration and one in validation sets, thereby producing correct classifications of 96% and 87% in each case. The most appealing results of the three datasets were that of SNV+2der‐treated spectra where 100% correct classification were obtained for calibration and prediction models. The positive impacts of these spectra‐filtering methods were apparent when comparing the discriminative capacities of untreated and treated spectral data as earlier supported (Hernández‐Martínez et al., 2013). However, the few misclassified samples (7%) in N and C classes did not significantly lower the coefficients of calibration and cross‐validation of the untreated spectral data. Similar results were obtained for untreated and treated spectra matrices with improved $R_{\mathrm{cal}}^{2}$ and $R_{\mathrm{cv}}^{2}$, when compared to model of chemical data (Table 3). It is noteworthy to state that both chemical and spectral data showed the same overall discriminative outcome for C palm oil samples (100%). Therefore, palm oils obtained from the North could only be completely separated when modeled using SNV+2der‐treated spectral data. Earlier observations showed better model outputs when SNV and second‐derivative spectra correction were applied simultaneously to spectroscopic data (Jolayemi et al., 2017).

**Table 2:** OPLS‐DA calibration and validation results: correct regional classification rates of the oils samples using quality parameters and spectral data

Data matrix	Member	OPLS‐DA Model			
		C	N	S	% CC
Quality parameters					
Calibration					
C	15	15	0	0	100
N	15	2	13	0	87
S	15	0	0	15	100
Total	45	16	14	15	96
Validation					
C	5	5	0	0	100
N	5	1	4	0	80
S	5	0	1	4	80
Total	15	6	5	4	87
UV‐vis_untreated					
Calibration					
C	15	15	0	0	100
N	15	1	14	0	93
S	15	0	0	15	100
Total	45	16	14	15	98
Validation					
C	5	5	0	0	100
N	5	1	4	0	80
S	5	0	0	5	100
Total	15	6	4	5	93
UV‐vis_SNV+2der					
Calibration					
C	15	15	0	0	100
N	15	0	15	0	100
S	15	0	0	15	100
Total	45	15	15	15	100
Validation					
C	5	5	0	0	100
N	5	0	5	0	100
S	5	0	0	5	100
Total	15	5	5	5	100

%CC: percentage of correct classification; C, central; N, North; S, South.

**Table 3:** OPLS‐DA calibration model performance parameters for chemical and spectral data matrices

Data matrix	PC_p + PC_o	$R_{\mathrm{cal}}^{2}$	$R_{\mathrm{cv}}^{2}$
Quality parameters	2 + 2	.86	.83
UV‐vis_Untreated	2 + 3	.94	.90
UV‐vis_SNV+2der	2 + 3	.95	.92

*PC_p + PC_o*: number of principal components (predictive+orthogonal); $R_{\mathrm{cal}}^{2}$: determination coefficient of calibration model; $R_{\mathrm{cv}}^{2}$: determination coefficient of leave‐one‐out cross‐validation model.

## 4 CONCLUSIONS

For the first time, application of UV‐visible spectroscopy and quality characteristics in geographical differentiation of palm oil was demonstrated. The models showed high potentials for regional recognition of palm oil when quality parameters, untreated and SNV+2der‐treated spectral data of the oils were elaborated using PCA and OPLS discriminant analysis. The performance of the models in terms of calibration and external prediction, percentage of correct classification, and coefficients of determinations (calibration and cross‐validation) was reasonably satisfactory in both spectral and chemical data. Application of spectra‐filtering algorithms significantly improved the discriminative capacity of the spectroscopic data. Models built on spectral data had higher coefficients of calibrations and cross‐validations with an average of 95%. The same inference was true when comparing the projection of observations in the score ellipses, between chemical and spectral data. However, both data were valuable discriminating tools effective in correctly classifying palm oils into their separate production regions with little intersection among class members. Finally, this analytical approach could represent a valid tool for the prevention of palm oil quality misrepresentation; a form of food fraud that may be prevalent in the country of high production. Speeds, straightforwardness, little to no sample alteration or treatment and less complicated equipment are few out of many advantages offer by spectroscopic method over usually expensive classical methods.

## CONFLICT OF INTEREST

The authors declare that they do not have any conflict of interest.

## ETHICAL REVIEW

This study does not involve any human or animal testing.

## INFORMED CONSENT

Written informed consent was obtained from all study participants.
